# Supplementary material for: Effects of thrombospondin-4 on pro-inflammatory phenotype differentiation and apoptosis in macrophages
Source: Cell Death Dis. 2020 Jan 23;11(1):53. doi: 10.1038/s41419-020-2237-2 (PMC6978349; doi:10.1038/s41419-020-2237-2)
Supplement: Supplementary file 1 — Supplementary Figures Legends [file 41419_2020_2237_MOESM1_ESM.docx]

**Supplemental Figures Legends**

**Suppl. Figure 1.**

Purity of mouse bone-marrow-derived macrophages (BMDM). BMDM were analyzed by flow cytometry using anti-CD11b and anti-F4/80 antibodies after differentiation in macrophage differentiation medium. 94 – 98 % of cultured cells were positive for both markers.

**Suppl. Figure 2. Dot blots showing individual data points for Fig. 2.**

**Suppl. Figure 3. Loading and transfer control for Fig. 2.**

Membranes used in Western blotting in Fig. 2 were stained with Ponceau Red to assure equal loading and transfer of protein.

**Suppl. Figure 4. Dot blots showing individual data points for Fig. 3.**

**Suppl. Figure 5. Dot blots showing individual data points for Fig. 4.**

**Suppl. Figure 6. Basal expression of markers of the pro-inflammatory macrophages in three genotypes.**

CD38 (A) and Nos2 (B) in BMDM from WT, *Thbs4^-/-^* (TSP4 KO), and CD38 expression in P387-TSP4-KI (TSP4 KI) mice (C).

**Suppl. Figure 7. Dot blots showing individual data points for Fig. 5.**

**Suppl. Figure 8. Dot blots showing individual data points for Fig. 6.**

**Suppl. Figure 9. Number of blood monocytes in WT (expressing A387-TSP4), *Thbs4^-/-^* (TSP4-KO), and P387-TSP4-KI (TSP4-KI) mice.**

**Suppl. Figure 10. Dot blots showing individual data points for Fig. 7.**
