# Supplementary figures and images for: Effects of thrombospondin-4 on pro-inflammatory phenotype differentiation and apoptosis in macrophages
Source: Cell Death Dis. 2020 Jan 23;11(1):53. doi: 10.1038/s41419-020-2237-2 (PMC6978349; doi:10.1038/s41419-020-2237-2)

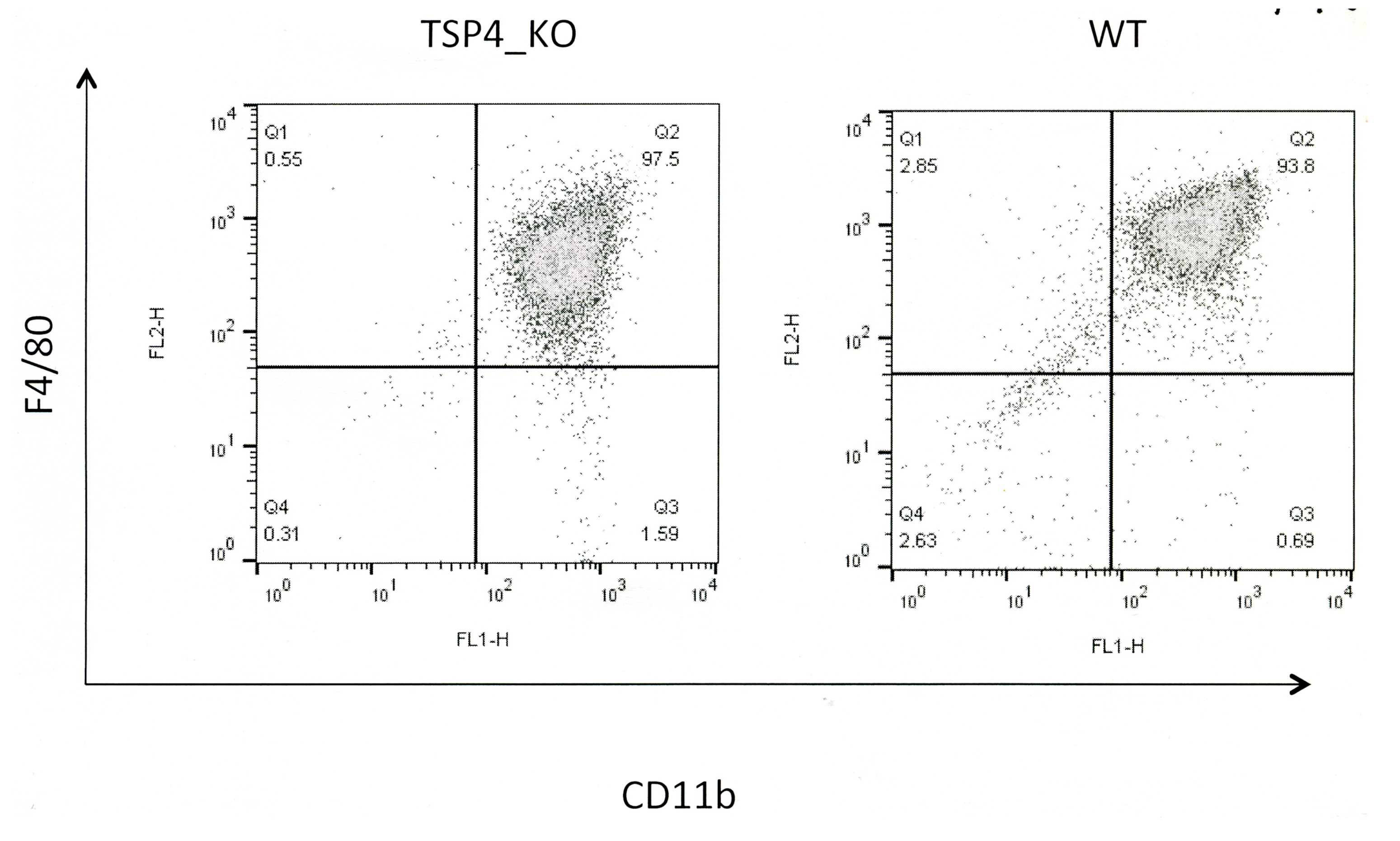

Supplement: Supplementary file 2 — Suppl. Fig.1 [file 41419_2020_2237_MOESM2_ESM.png]

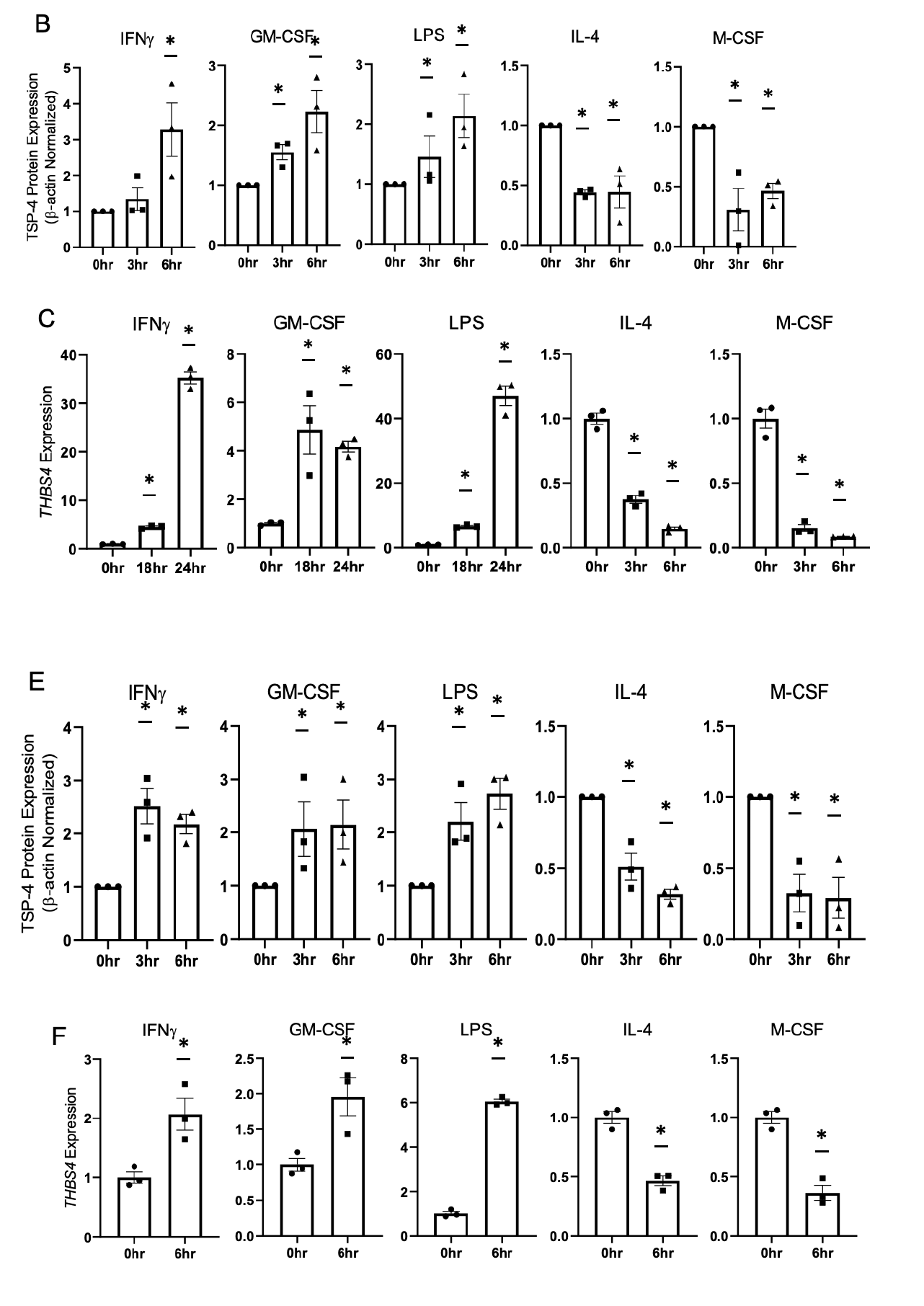

Supplement: Supplementary file 3 — Suppl. Fig.2 [file 41419_2020_2237_MOESM3_ESM.png]

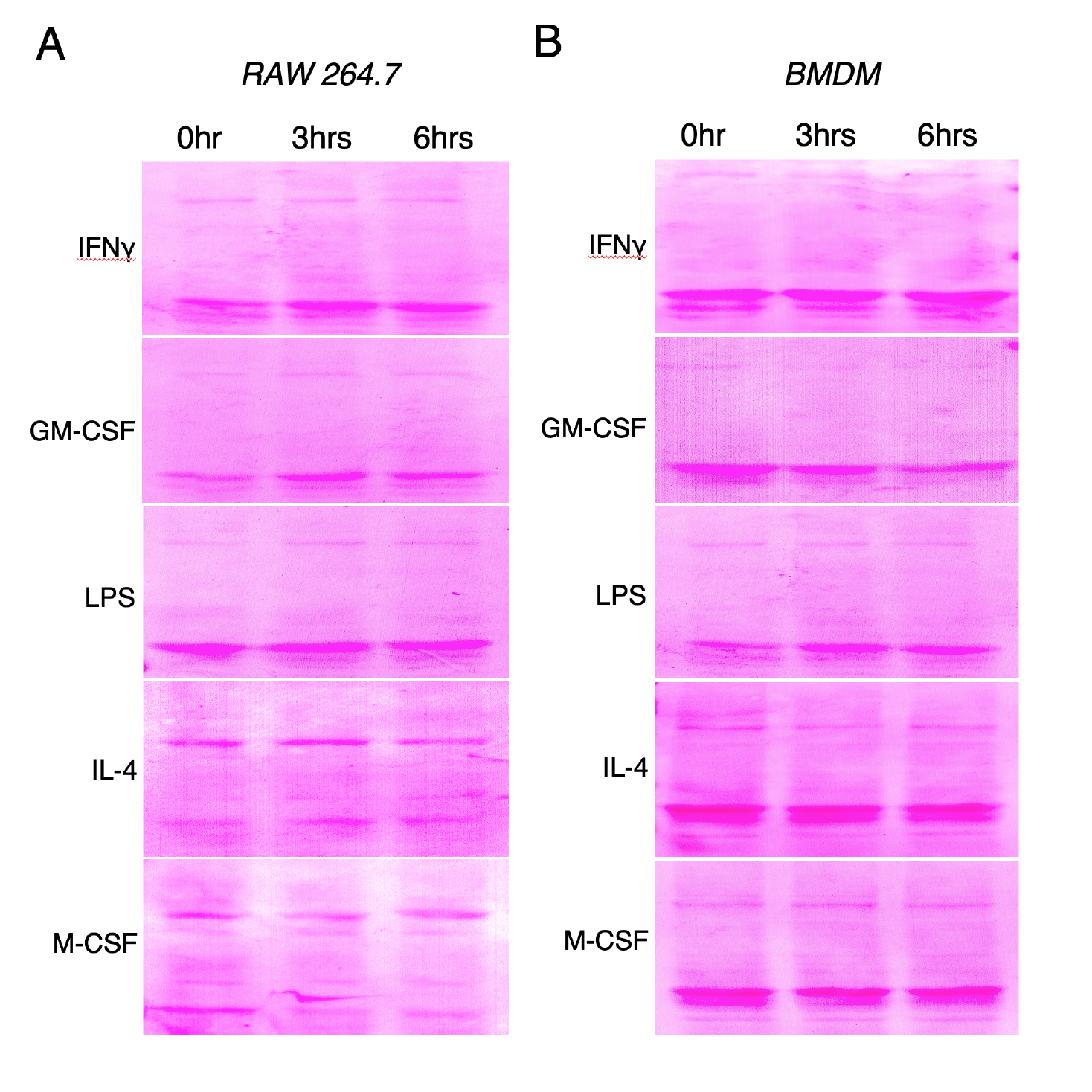

Supplement: Supplementary file 4 — Suppl. Fig.3 [file 41419_2020_2237_MOESM4_ESM.png]

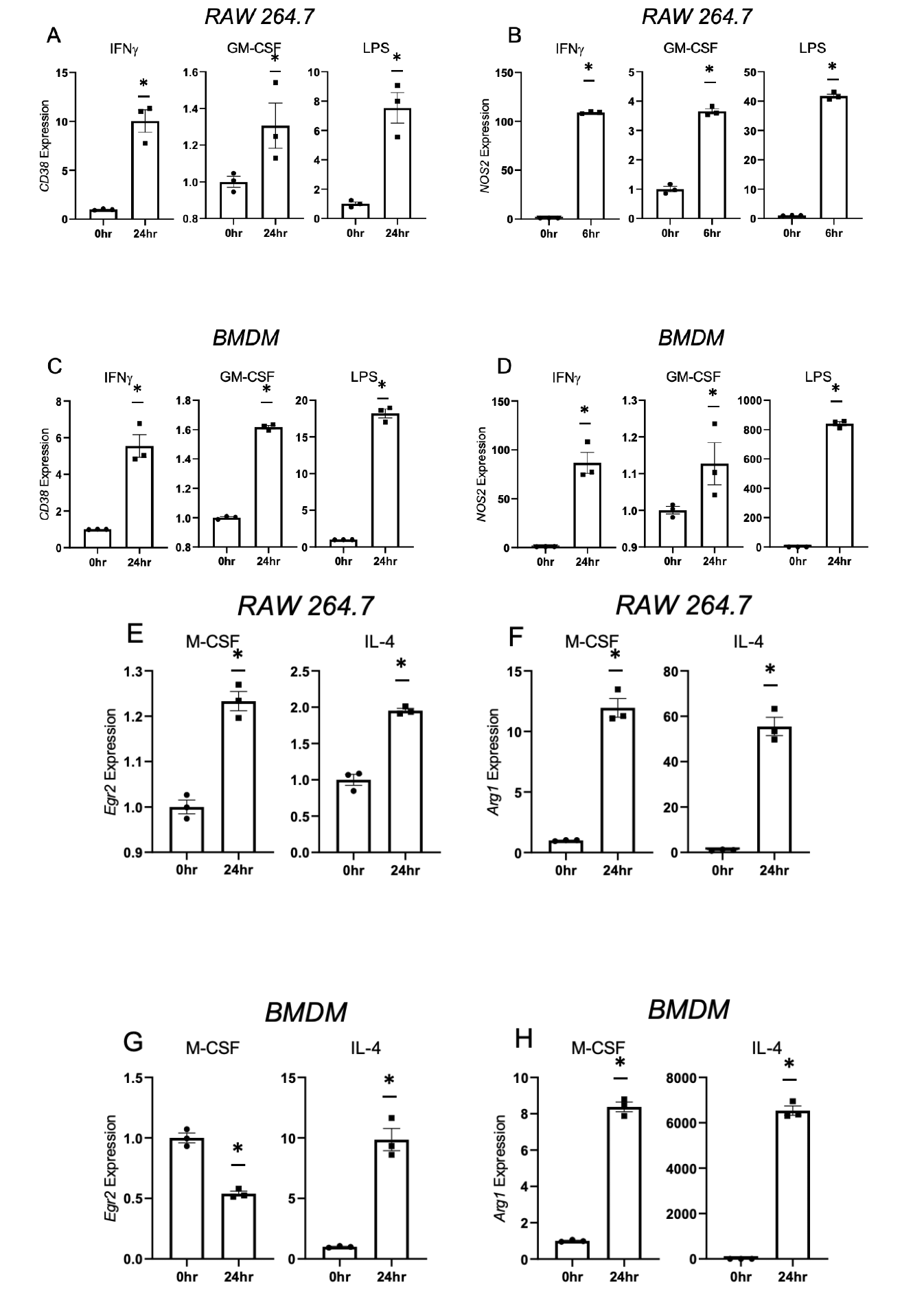

Supplement: Supplementary file 5 — Suppl. Fig.4 [file 41419_2020_2237_MOESM5_ESM.png]

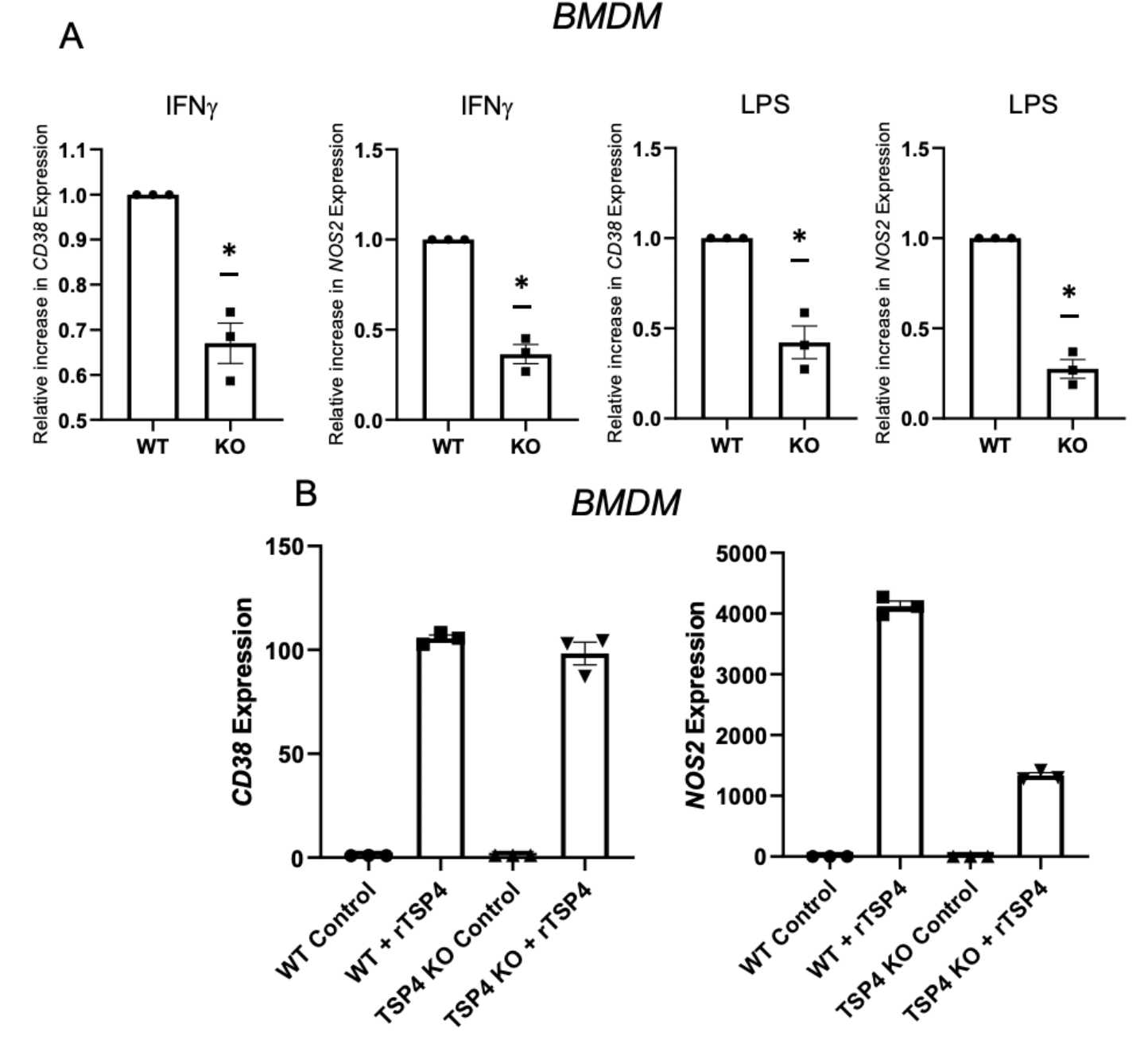

Supplement: Supplementary file 6 — Suppl. Fig.5 [file 41419_2020_2237_MOESM6_ESM.png]

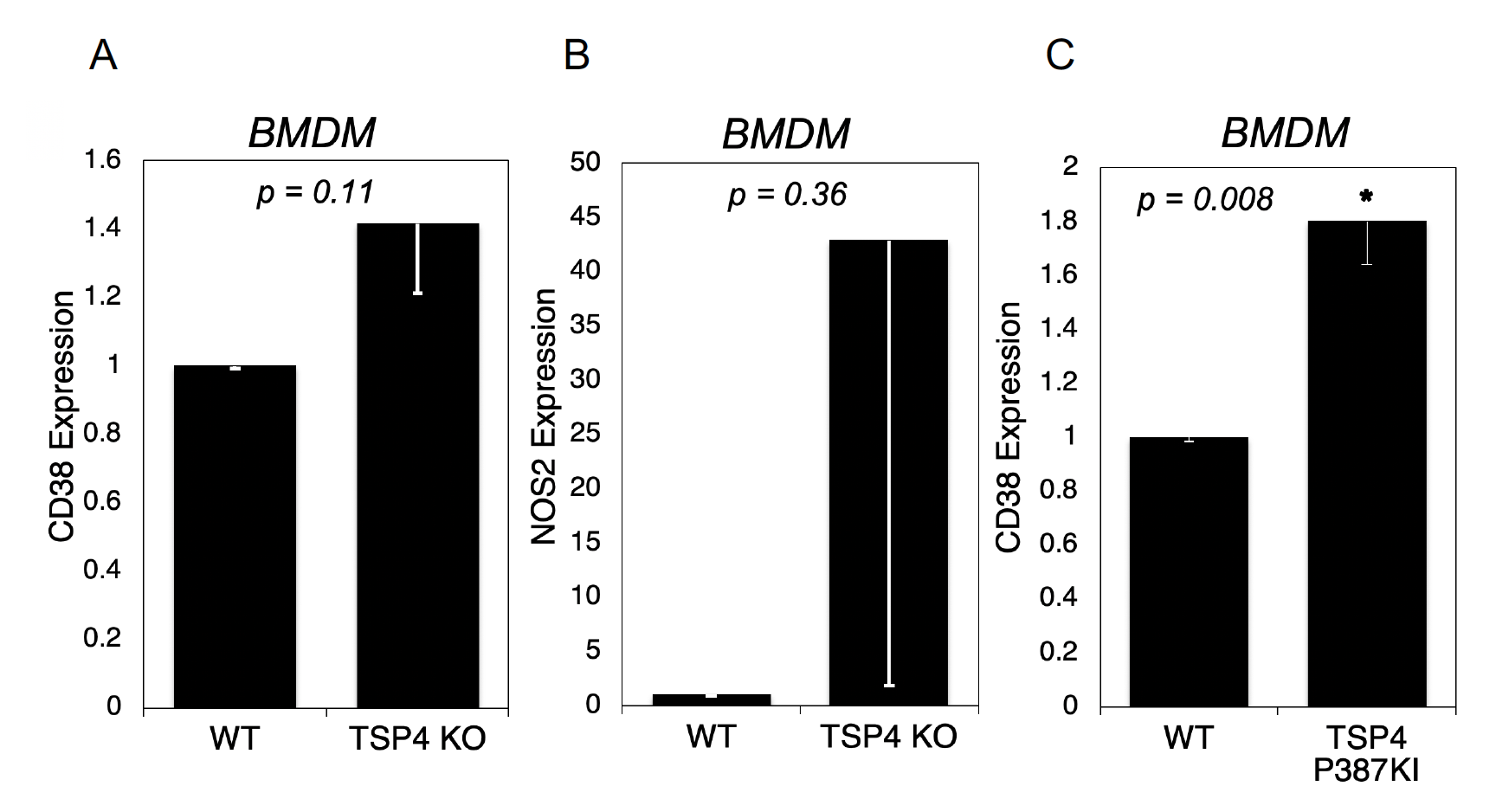

Supplement: Supplementary file 7 — Suppl. Fig.6 [file 41419_2020_2237_MOESM7_ESM.png]

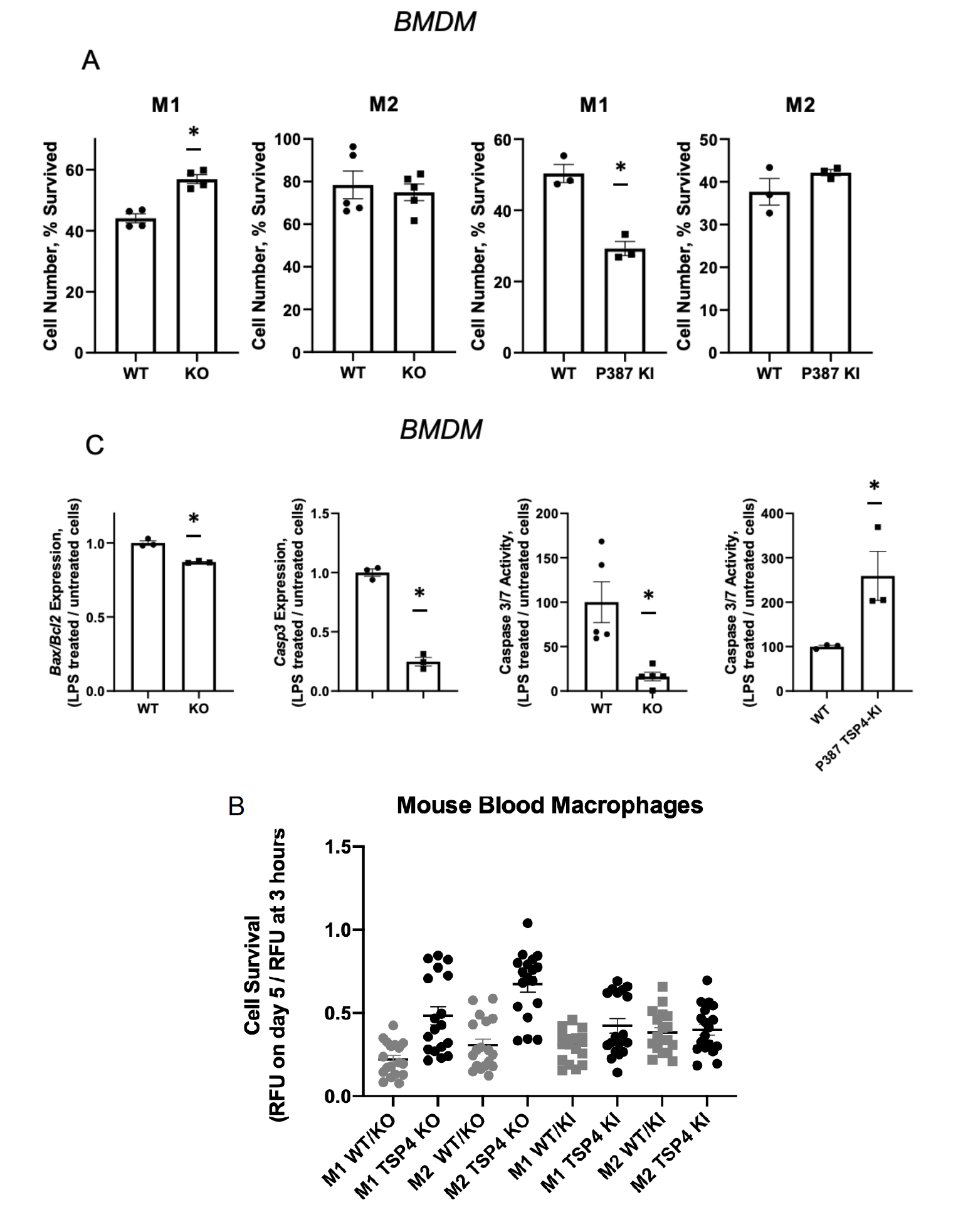

Supplement: Supplementary file 8 — Suppl. Fig.7 [file 41419_2020_2237_MOESM8_ESM.png]

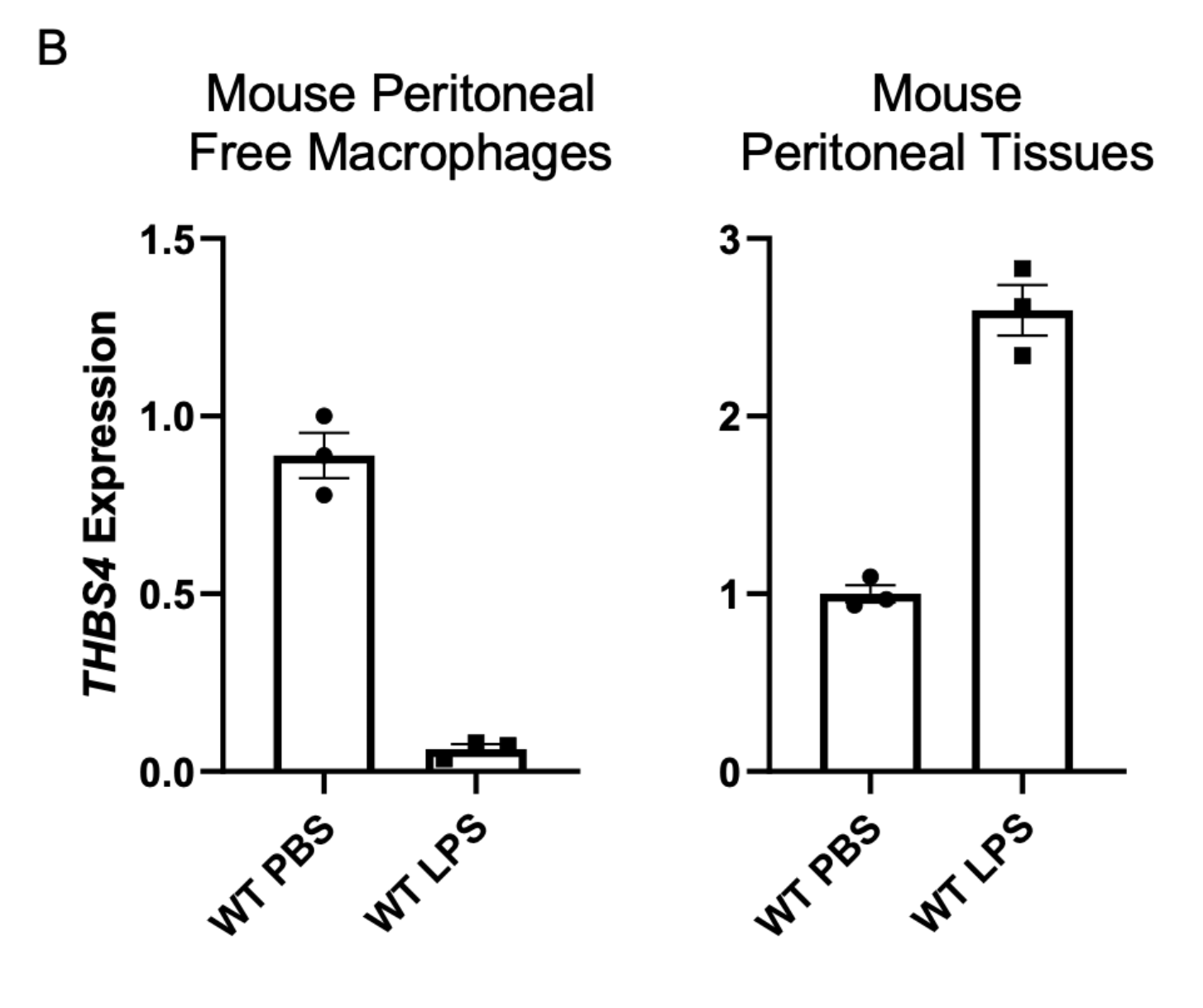

Supplement: Supplementary file 9 — Suppl. Fig.8 [file 41419_2020_2237_MOESM9_ESM.png]

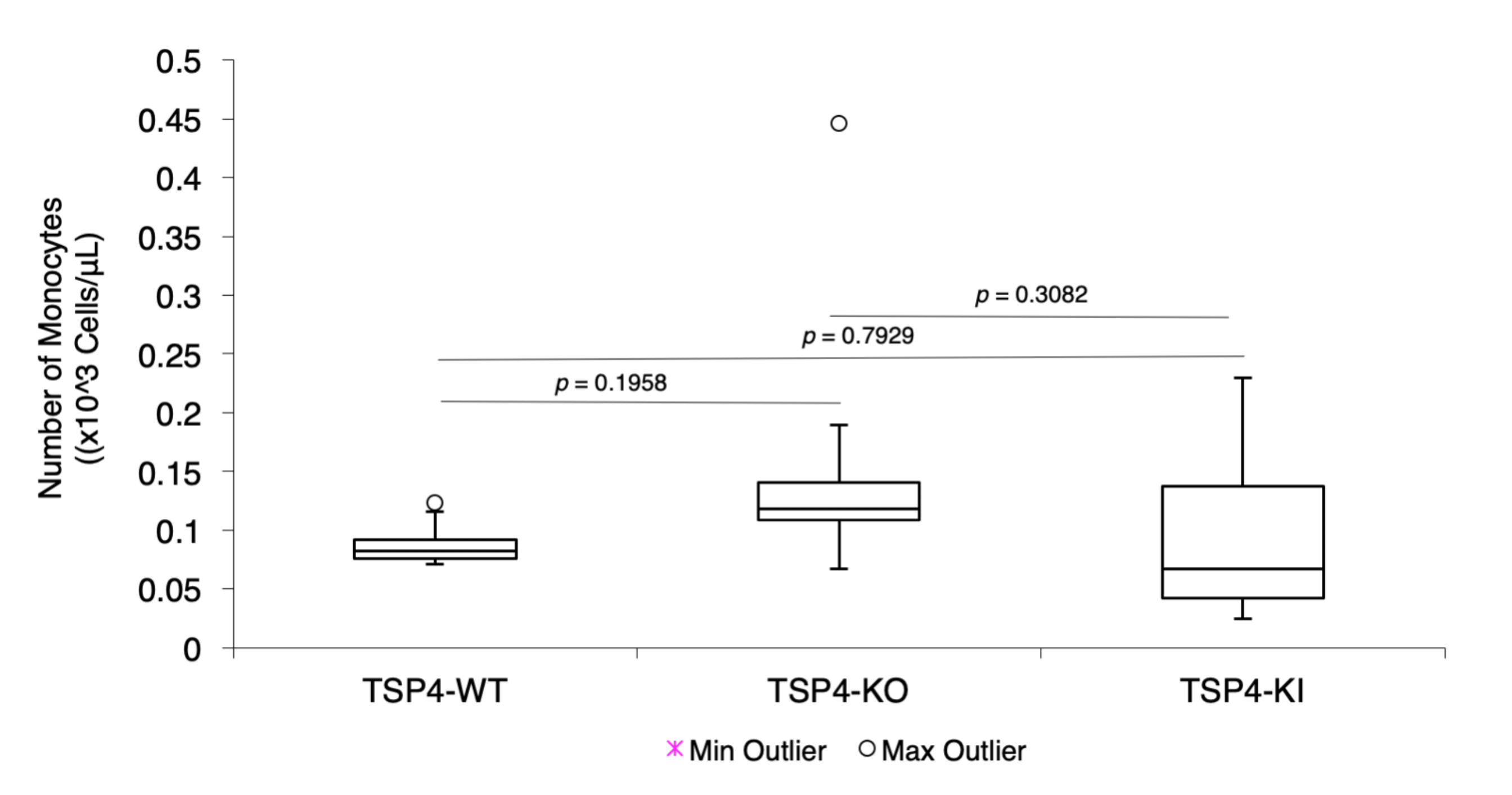

Supplement: Supplementary file 10 — Suppl. Fig.9 [file 41419_2020_2237_MOESM10_ESM.png]

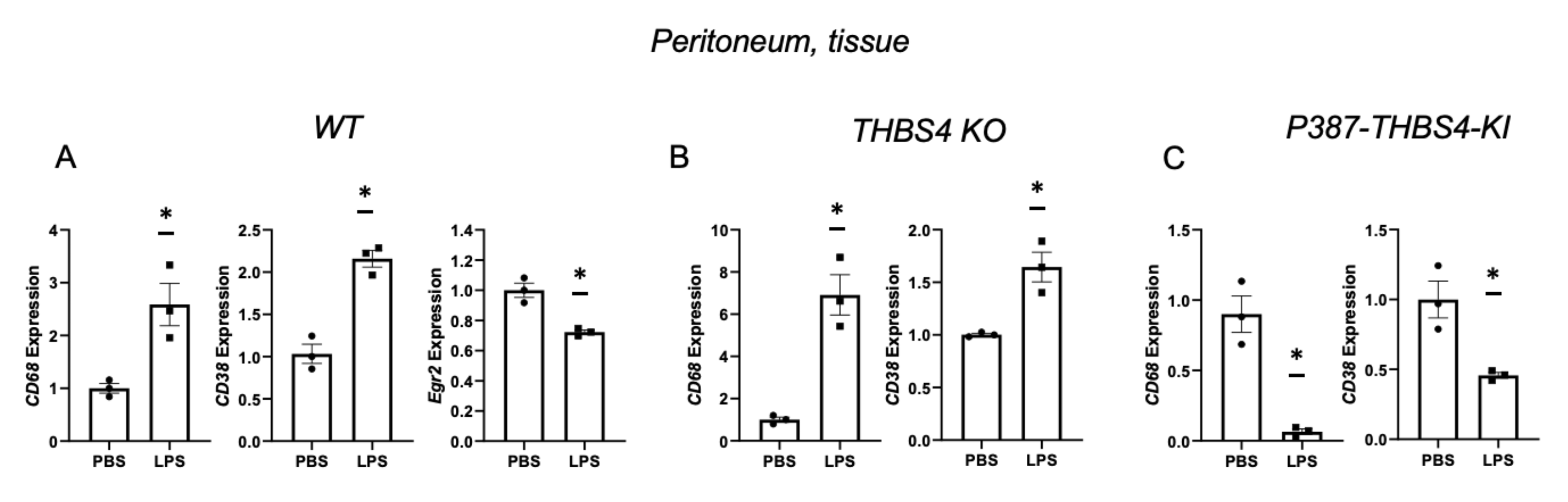

Supplement: Supplementary file 11 — Suppl. Fig.10 [file 41419_2020_2237_MOESM11_ESM.png]
